# Managing the positive periosteal margin after Mohs excision of scalp dermatofibrosarcoma protuberans in children with underlying calvarial bone thinning and resorption: The value of multidisciplinary treatment

**DOI:** 10.1016/j.jdcr.2023.06.044

**Published:** 2023-07-13

**Authors:** Eugene Zheng, Sai Cherukuri, Christopher J. Arpey, Edward S. Ahn, Samir Mardini, Waleed Gibreel

**Affiliations:** a Division of Plastic and Reconstructive Surgery, Department of Surgery, Mayo Clinic, Rochester, Minnesota; b Division of Dermatology, Mayo Clinic, Rochester, Minnesota; c Division of Neurosurgery, Mayo Clinic, Rochester, Minnesota

**Keywords:** calvarial reconstruction, cranioplasty, dermatofibrosarcoma protuberans, Mohs micrographic surgery

## Introduction

Dermatofibrosarcoma protuberans (DFSP) affecting the scalp often disfigures the shape of the head but is also known to extend intracranially to cause neurologic symptoms if not addressed in time.^1^ When the periosteal margin is involved, deep margin clearance requires addressing the underlying bone.^2^ In these circumstances and in the absence of intracranial extension, the outer table of the skull is usually “burred down” to achieve a negative margin. This approach is feasible in older patients with a thick calvarial bone. However, in children, the underlying calvarial bone can be quite thin, and any attempt at scraping or “burring down” the bone can easily create a full-thickness calvarial defect. These full-thickness calvarial defects can be problematic especially when they are located over major venous sinuses. An attempt to burr down or scrape such thin bones may result in an inadvertent injury to the dural sinuses. To achieve a negative margin in this setting, craniectomy may be needed.

We describe the case of a 6-year-old girl with DFSP of the scalp, involving the periosteum, which was treated with a single-stage surgery involving a multidisciplinary team comprising of a Mohs micrographic surgeon, pediatric neurosurgeon, and pediatric plastic surgeon.

## Case report

**Fig 1:**
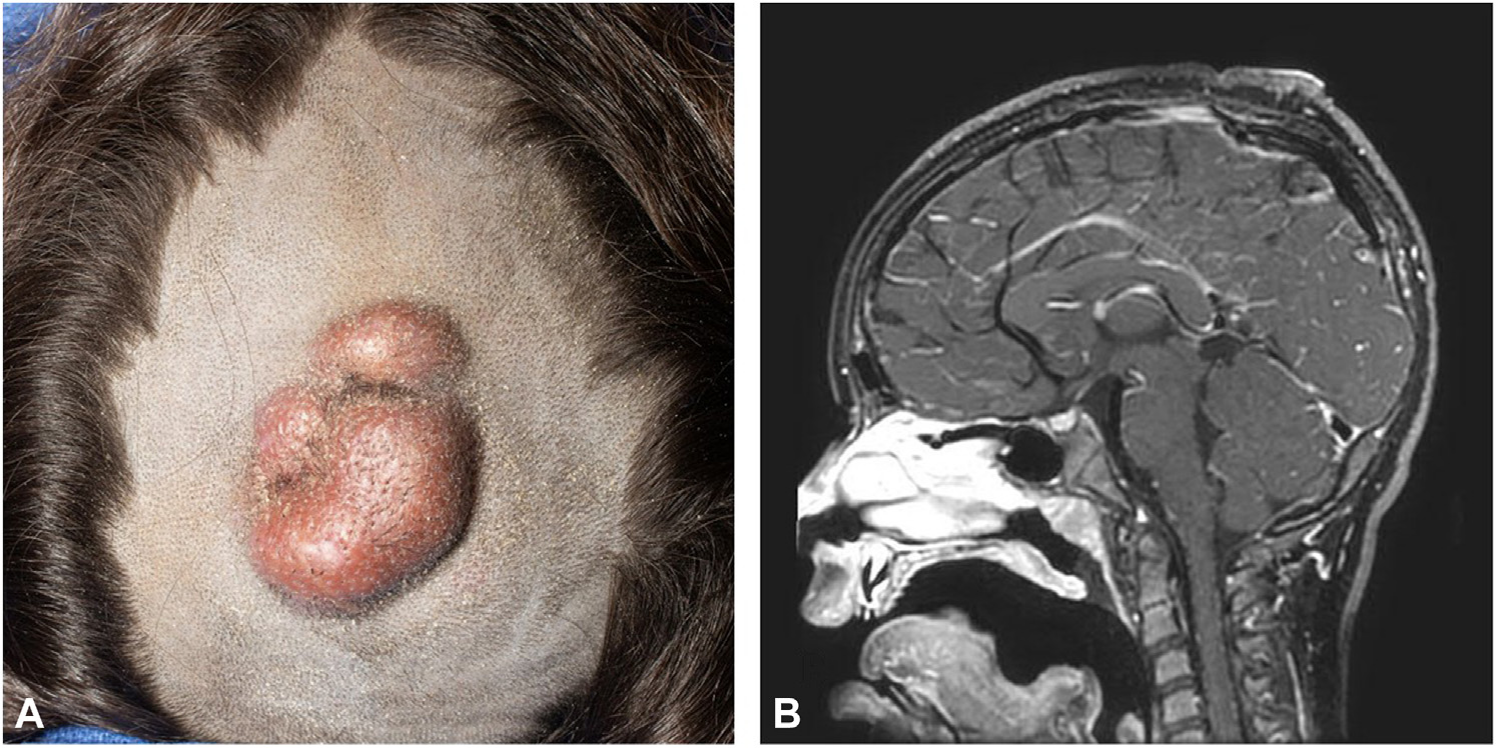
A, Presurgical assessment showing a 5 × 4-cm^2^ mass with a satellite nodule on the scalp of the patient. **B,** Magnetic resonance imaging of the brain, with the sagittal view showing osseous remodeling of the calvarium in the region of the tumor on the vertex. Note that there was no evidence of intracranial extension.

A 6-year-old girl presented with a tender midline lesion, measuring 5 × 4 cm, on the vertex (Fig 1, *A*). It was initially noticed at birth as a depression in the skin a few millimeters in diameter. However, at the age of 3 years, the patch grew, prompting an ultrasound scan, which showed normal underlying bone. By the age of 5 years, the lesion had grown precipitously, becoming nodular and painful, with secondary local satellite lesions. The patient did not have any neurologic symptoms. Multiple punch biopsies showed spindle cell proliferation, consistent with DFSP. Platelet-derived growth factor translocation was notably negative. A chest x-ray was performed and did not show any concern for metastatic disease. There were no other symptoms or signs suggestive of metastatic spread.

Computed tomography and magnetic resonance imaging of the head revealed a 3.5 cm × 4.7 cm × 7-mm region of ill-defined soft tissue involving the skin and subcutaneous tissue, interdigitating with the underlying musculature. Although there was no obvious intracranial extension, it was noted that there was bilateral smooth osseous thinning of the bilateral frontal and parietal bones overlying the sagittal venous sinus (Fig 1, *B*). These findings raised our suspicion for possible periosteal and/or underlying bone involvement. To holistically address the diagnosis, a multidisciplinary team of a Mohs micrographic surgeon, pediatric neurosurgeon, and pediatric plastic surgeon was formed for excision of the mass and reconstruction of the calvarium and scalp.

**Fig 2:**
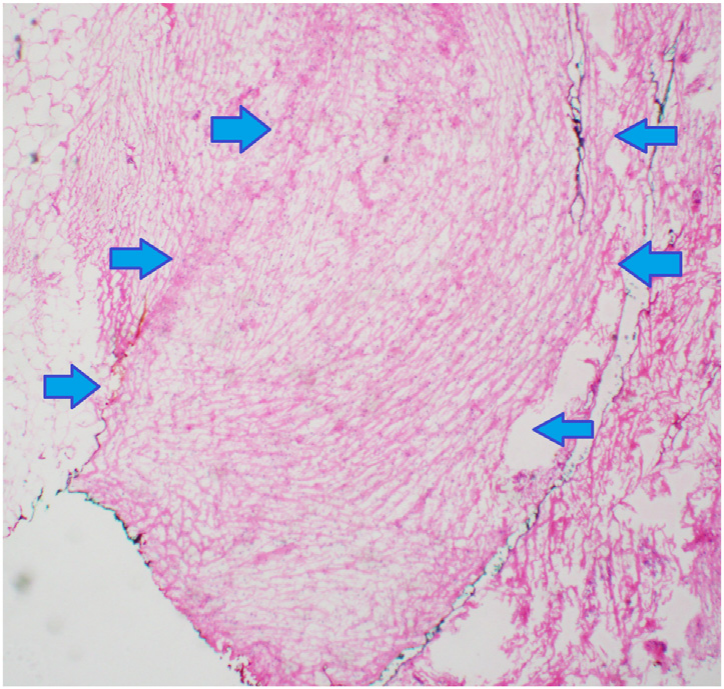
Representative hematoxylin and eosin (4×) Mohs micrographic section from stage 1 of Mohs micrographic surgery with hypercellular fibroblasts with galea aponeurotica outlined by arrows consistent with dermatofibrosarcoma protuberans. Normal adipose overlying galea is present on the left of arrow tails. Normal cellularity of unaffected fascia is present on the right of the arrow tails.

**Fig 3:**
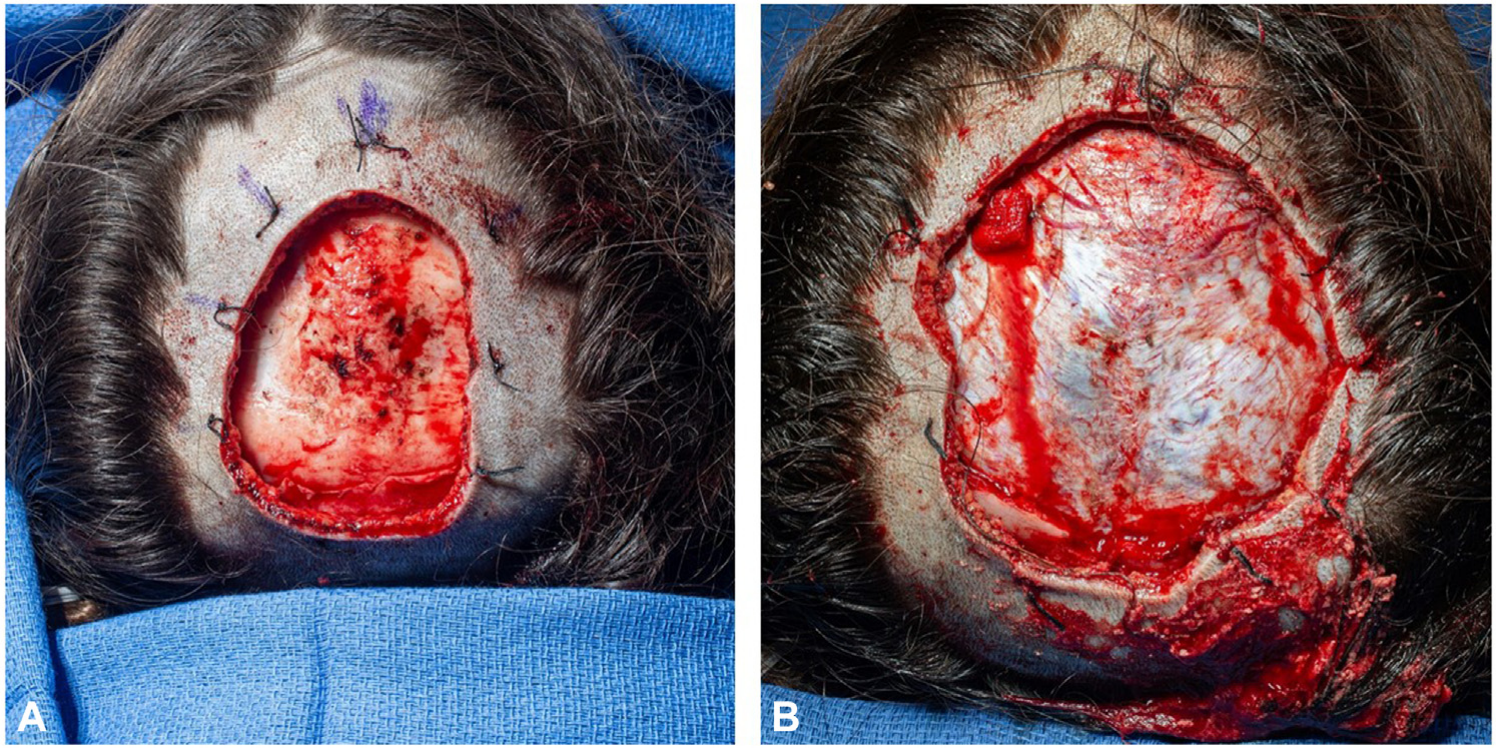
View of the scalp after (**A**) the Mohs micrographic surgery procedure and (**B**) craniectomy. The skin defect measured ∼8 × 12 cm. The bony defect measured ∼8 × 11 cm.

Mohs micrographic surgery proceeded initially with the patient under general anesthesia. Cutaneous free margins were obtained after the fourth excision. The tumor was noted to have subclinical and microscopic extension within the galea and periosteum (Fig 2). The calvarium was found to be grossly pitted centrally (Fig 3, *A*). Because of periosteal involvement of the tumor, a craniotomy was performed by the neurosurgeon. The bone flap started at the bregma and extended posterior over the midline. This was completed without complication, and no dural tears were made (Fig 3, *B*).

**Fig 4:**
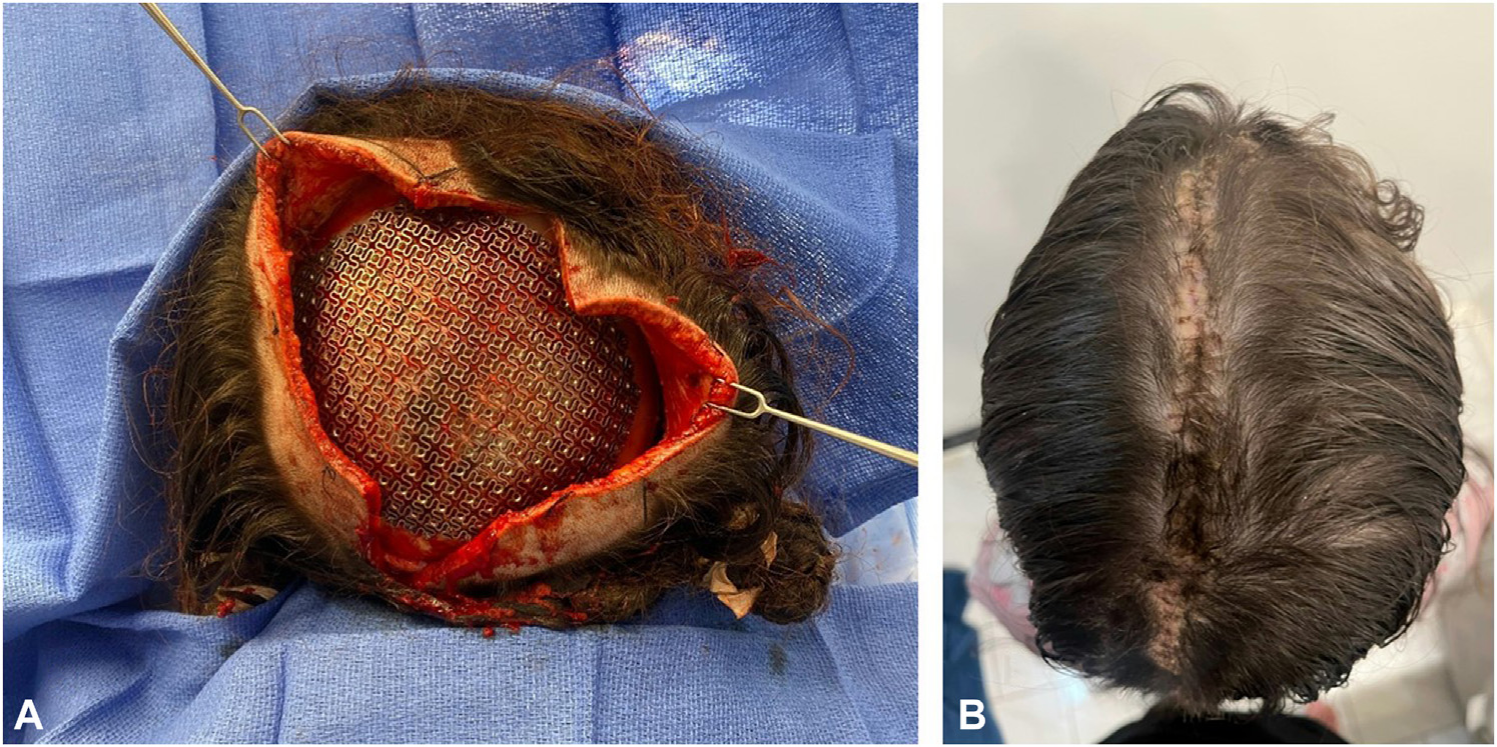
Reconstruction of the defect. **A,** Placement of a titanium mesh for calvarial reconstruction. **B,** Postoperative appearance at 9 months after scalp local flap reconstruction

Following these procedures, a full-thickness scalp defect measuring 8 × 12 cm and a bone defect measuring 8 × 11 cm were noted. A titanium mesh spanning the cranial defect was placed and secured in place using screws (Fig 4, *A*). Galeal scoring and circumferential subgaleal undermining allowed advancement of the scalp toward the midline. Two back cuts in a yin-yang fashion allowed further advancement. Intraoperative tissue expansion was performed by placement of tissue expanders on both sides, resulting in a gain of at least 1 cm on each side, allowing tension-free approximation across the midline. A segment of the scalp tissue that was missing galea was excised to allow continuity in the galea (Fig 4, *B*).

The patient recovered from the operation uneventfully and was discharged on postoperative day 4. Final soft-tissue pathology showed margin-free excision and a grossly indented calvarium bone but no obvious evidence of bony DFSP invasion. There were no wound healing issues, and the patient remained disease free at her 19-month follow-up visit, as determined based on clinical examinations. Given the high confidence in complete resection, no additional follow-up has been scheduled or arranged.

## Discussion

Dermatofibrosarcoma of the scalp has historically shown potential for periosteum invasion. Although only a few cases of this have been reported, craniectomy is typically required for establishment of margin-free resection.^3^ Given the location of the lesion over the sagittal dural venous sinus and significant thinning of the underlying bone, a multidisciplinary decision was made to proceed with craniectomy to achieve a negative margin following Mohs micrographic surgery.

Because calvarial growth is usually complete at ∼5 years of age, titanium implants and other prosthetic materials should not affect calvarial growth.^4^^,^^5^ When calvarial reconstruction is needed in younger patients, our preference is to use autologous, calvarial grafts as long as the calvarial bone allows splitting of the outer and inner tables (usually feasible in patients aged >1 year). If prosthetic implants are used in patients aged <5 years, we expect the implants to contribute to some growth abnormalities. In these situations, the families are counseled on the possibility of needing future revisions as the child’s head continues to grow.

Despite preoperative imaging and gross appearance of the bone at the time of resection raising suspicion for underlying calvarium involvement, the final pathology did not show any bone invasion. The authors believe that pursuing craniectomy was critical in achieving the optimal outcome for our patient even if the final pathology did not confirm bony involvement. With this approach, we have high confidence in our surgical margins in a disease that is notorious for a high recurrence rate. Furthermore, resection and reconstruction were conducted under a single session of general anesthesia and limited additional trips to the operating room.

The formation of the multidisciplinary team was instrumental for the management of this patient. The recurrent nature of the lesion, location being in a sensitive spot over the dural sinuses, and desire for single-stage hard- and soft-tissue reconstruction necessitated the expertise of each member of the team. Therefore, we believe that multidisciplinary referral to a tertiary center with the availability of a Mohs micrographic surgeon, pediatric neurosurgeon, and pediatric plastic surgeon is advised to allow single-stage, margin-free resection and immediate reconstruction.

## Conflicts of interest

None disclosed.
